# Comparison of antigen and antibody responses in repeat lymphatic filariasis transmission assessment surveys in American Samoa

**DOI:** 10.1371/journal.pntd.0006347

**Published:** 2018-03-09

**Authors:** Kimberly Y. Won, Keri Robinson, Katy L. Hamlin, Joseph Tufa, Margaret Seespesara, Ryan E. Wiegand, Katherine Gass, Joseph Kubofcik, Thomas B. Nutman, Patrick J. Lammie, Saipale Fuimaono

**Affiliations:** 1 Centers for Disease Control and Prevention, Division of Parasitic Diseases and Malaria, Atlanta, GA, United States of America; 2 Swiss Tropical and Public Health Institute, Epidemiology and Public Health, Basel, Switzerland; 3 University of Basel, Tropical and Public Health Sciences, Basel, Switzerland; 4 Department of Health, Lymphatic Filariasis Elimination Program, Pago Pago, American Samoa; 5 Task Force for Global Health, Neglected Tropical Diseases Support Center, Decatur, GA, United States of America; 6 National Institutes of Health, National Institute of Allergy and Infectious Diseases, Bethesda, MD, United States of America; Erasmus MC, NETHERLANDS

## Abstract

**Background:**

Current WHO recommendations for lymphatic filariasis (LF) surveillance advise programs to implement activities to monitor for new foci of transmission after stopping mass drug administration (MDA). A current need in the global effort to eliminate LF is to standardize diagnostic tools and surveillance activities beyond the recommended transmission assessment survey (TAS).

**Methodology:**

TAS was first conducted in American Samoa in 2011 (TAS 1) and a repeat TAS was carried out in 2015 (TAS 2). Circulating filarial antigen (CFA) and serologic results from both surveys were analyzed to determine whether interruption of LF transmission has been achieved in American Samoa.

**Principal findings:**

A total of 1,134 and 864 children (5–10 years old) were enrolled in TAS 1 and TAS 2, respectively. Two CFA-positive children were identified in TAS 1, and one CFA-positive child was identified in TAS 2. Results of both surveys were below the threshold for which MDA was warranted. Additionally, 1,112 and 836 dried blood spots from TAS 1 and TAS 2, respectively were tested for antibodies to Wb123, Bm14 and Bm33 by luciferase immunoprecipitation system (LIPS) assay and multiplex bead assay. In 2011, overall prevalence of responses to Wb123, Bm14, and Bm33 was 1.0%, 6.8% and 12.0%, respectively. In 2015, overall prevalence of positive Bm14 and Bm33 responses declined significantly to 3.0% (p<0.001) and 7.8% (p = 0.013), respectively.

**Conclusions/Significance:**

Although passing TAS 1 and TAS 2 and an overall decline in the prevalence of antibodies to Bm14 and Bm33 between these surveys suggests decreased exposure and infection among young children, there were persistent responses in some schools. Clustering and persistence of positive antibody responses in schools may be an indication of ongoing transmission. There is a need to better understand the limitations of current antibody tests, but our results suggest that serologic tools can have a role in guiding programmatic decision making.

## Author summary

Lymphatic filariasis (LF), endemic in 72 countries, is a debilitating mosquito-transmitted parasitic disease caused by filarial worms. The Global Program to Eliminate Lymphatic Filariasis (GPELF) aims to interrupt transmission through mass drug administration (MDA) and to reduce suffering caused by the disease. At the start of GPELF in 2000 it was estimated that approximately 1.4 billion people were at risk for infection. By the end of 2016, primarily through successful MDA programs, the global number of people requiring interventions was reduced to 856.4 million. Current recommendations by the World Health Organization for LF surveillance advise programs to implement activities to monitor for new foci of transmission after stopping MDA. A current need in the global effort to eliminate LF is to standardize diagnostic tools and surveillance activities beyond the recommended transmission assessment survey (TAS). Two TAS were conducted in American Samoa; first in 2011 (TAS 1) and repeated in 2015 (TAS 2). In our evaluation, circulating filarial antigen and serologic results from both surveys were analyzed to determine whether interruption of LF transmission has been achieved in American Samoa. Despite passing TAS 1 and TAS 2, clustering and persistence of positive antibody responses in schools may be an indication of ongoing transmission. Results from our evaluation suggest that serologic tools can have a role in guiding programmatic decision-making.

## Introduction

Lymphatic filariasis (LF), endemic in 72 countries, is a debilitating mosquito-transmitted parasitic disease caused by filarial worms (*Wuchereria bancrofti* and *Brugia* spp.) [1]. In 1997, at the 50^th^ World Health Assembly (WHA), a resolution was passed to eliminate LF as a public health problem by 2020 [2]. Shortly thereafter, in 1999, the Pacific Program for the Elimination of Lymphatic Filariasis (PacELF) was established to eliminate the disease in the Pacific Region through a strategy of annual rounds of mass drug administration (MDA) [3]. The following year, the Global Program to Eliminate Lymphatic Filariasis (GPELF) was established to assist all LF-endemic countries in achieving this elimination goal through the same MDA strategy. At the start of GPELF it was estimated that approximately 1.4 billion people were at risk for infection. By the end of 2016, MDA had been implemented in 66 of 72 LF-endemic countries, with a cumulative total of 6.7 billion treatments delivered since the start of GPELF [4].

After multiple rounds of MDA, LF elimination programs must be able to determine when it is appropriate to stop treatment. The World Health Organization (WHO)-recommended transmission assessment survey (TAS) was designed as a decision-making tool to determine when transmission of LF is presumed to have reached a level low enough that it cannot be sustained even in the absence of MDA [5]. In areas where *W*. *bancrofti* is the principal LF pathogen, infection is assessed in the TAS by measuring circulating filarial antigen (CFA). Since its integration into national programs in 2011, TAS has successfully been implemented across LF endemic countries, and based on the results, MDA has been discontinued in multiple locations. The global number of people requiring MDA has been reduced from 1.4 billion in 2000 to 856.4 million in 2016 [4].

Effective monitoring and evaluation (M&E) is not only necessary during the MDA period but important throughout the lifespan of the LF program, including after MDA has stopped. Current WHO recommendations for post-MDA surveillance include periodic surveys: repeating TAS twice at 2- to 3-year intervals after stopping MDA. Beyond the TAS, post-MDA surveillance guidance has not been standardized. Current WHO recommendations for surveillance advise programs to implement activities to monitor for new foci of transmission through the assessment of microfilaremia, antigenemia, or antibodies [5]. After effective MDA, microfilaremia and antigenemia begin to decline in populations and become increasingly difficult to detect [6]. Detection of antifilarial antibodies appears to provide the earliest indicator of filarial exposure [7], and the absence of detectable antibody responses may provide evidence that transmission has been interrupted.

Surveys conducted in 1999 indicated that 17% of residents in 18 villages in American Samoa were infected with *W*. *bancrofti* [3]. This established American Samoa as one of the areas with the highest filarial infection levels in the Pacific Region and the only U.S. territory endemic for LF. The American Samoa Department of Health (DOH) started MDA in 2000. Annual MDA coverage was low (<50%) prior to 2003. After reassessment and modification of the communication and distribution strategies, the program treated an estimated 70% and 65% of the population in 2003 and 2004, respectively [8]. Results from surveys in four sentinel sites showed an overall decline in CFA levels from 13% in 2003 to 0.95% in 2006 [9]. An island-wide survey was conducted in 2007, and CFA prevalence was 2.3%, with the majority of the antigenemia detected in adults [10]. Because LF was presumed to be at very low levels, minimal programmatic activities were conducted from 2008–2010.

In accordance with WHO recommendations, TAS 1 was conducted in American Samoa in 2011 and was repeated in 2015 (TAS 2). The DOH opted to include antifilarial antibody testing in both surveys to complement antigen testing. In this paper we report CFA and serologic results from the two TAS that were conducted to determine whether or not interruption of LF transmission has been achieved in American Samoa.

## Methods

### Ethics statement

The surveys were approved by the DOH Institutional Review Board (IRB) and the U.S. Centers for Disease Control and Prevention (CDC) as program evaluation, non-research. In preparation for the TAS, survey details were described in a written document distributed to school officials and parents or guardians of potential participants. In accordance with DOH and Department of Education policies, parents or guardians provided written permission for participation of children. Additionally, children ≥7 years of age were asked to provide oral assent for their participation on the day of the survey. All data were collected electronically, and identifiable information was kept confidential and maintained by using a secure database with access restricted to essential survey personnel.

### Survey site and design

American Samoa, a U.S. territory, is located in the South Pacific comprising of seven small islands and atolls. More than 90% of the total population live on the main island of Tutuila with the remainder of the residents dispersed on the adjacent island of Aunu’u and the outer Manu’a islands of Ta’u, Ofu and Olosega. Tutuila and Aunu’u comprised the evaluation unit for TAS. TAS 1 was carried out in February 2011 and TAS 2 was conducted in April 2015. Surveys were implemented according to WHO guidelines for conducting TAS in areas where *Aedes* spp. are the main LF vectors [5]. Because of high school enrollment rates (>95%), school-based surveys were conducted at both time points, and grades 1 and 2 were used as a proxy for the recommended age (6–7 years). Systematic sampling was recommended for both surveys, but due to low rates of consent, all children with signed consent forms were enrolled. The target sample sizes in 2011 and 2015 were 1,042 and 1,014, respectively. The critical cutoff, the maximum number of observed positive results that is consistent with a threshold of < 1%, for both surveys was six antigen-positive children.

### Blood collection and examination

For both surveys approximately 160 μL of blood was collected via a single finger stick into an EDTA-coated blood collection tube (Ram Scientific, Yonkers, NY). One hundred microliters of blood was used for the detection of CFA by immunochromatographic card test (ICT) (Alere; Scarborough, ME). The cards were read at 10 min and marked as either positive or negative according to the manufacturer’s instructions. The remaining 60 μL of blood (10 μL per extension x 6 extensions) was spotted onto filter paper (Cellabs, Sydney, Australia), dried and stored at -20°C until shipped to National Institutes of Health (NIH) for antifilarial antibody testing by luciferase immunoprecipitation system (LIPS) assay [11] or CDC for testing by multiplex bead assay (MBA) [12–14] (described below).

### LIPS

In TAS 1, IgG responses to Wb123 were determined by previously described LIPS assay [11]. One modification was made to accommodate the use of dried blood spots (DBS) instead of serum. DBS were eluted in 200 μl of PBS, and 40 μl of the eluted material was used for the assay. Cutoff values were calculated from receiver operator characteristic (ROC) curves using sera from *W*. *bancrofti*-infected patients and presumed negative sera from North Americans with no history of foreign travel.

### MBA

Antifilarial antibody responses to Bm14 [15] and Bm33 [16] for samples collected during TAS 1 and responses to Wb123, Bm14 and Bm33 for samples collected during TAS 2 were determined by previously described MBA [7, 12–14]. Briefly, DBS were eluted to yield a sample dilution of 1:400 in PBS buffer (pH 7.2) containing 0.3% Tween-20, 0.02% sodium azide, 0.5% casein, 0.5% polyvinyl alcohol (PVA), 0.8% polyvinylpyrrolidone (PVP), and 3 μg/ml *Escherichia coli* extract. *E*. *coli* extract was added to the buffer to absorb antibodies to any residual *E*. *coli* proteins that may not have been eliminated in the antigen purification process. Samples having a coefficient of variation of >15% between duplicate wells for ≥2 positive LF antibody responses were repeated. The average of the median fluorescent intensity (MFI) values from the duplicate wells minus the background (bg) fluorescence from the buffer-only blank was reported as MFI-bg. Cutoff values were calculated from ROC curves using sera from *W*. *bancrofti*-infected patients and presumed negative sera from US citizens with no history of foreign travel.

### Treatment

Parents or guardians of individuals who were ICT positive were notified of test results, and the children were offered a standard single dose of diethylcarbamazine (DEC) (6 mg/kg) and albendazole (400 mg).

### Statistical analysis

Analyses were performed in R version 3.3.0 [17] with the survey package [18] using a 5% level of significance. Because a high percentage of the American Samoa population of 1^st^ and 2^nd^ graders participated, samples from TAS 1 and TAS 2 were treated as clustered samples with a finite population correction. Differences in frequencies were evaluated with a Rao-Scott Χ^2^ statistic [19]. Confidence intervals for proportions utilize the incomplete beta function [20]. Changes in MFI were evaluated with the complex sampling version of Mood’s test for differences in medians [21].

## Results

### TAS 1

A total of 1,134 children from 25 of 26 public and private elementary schools were enrolled in TAS 1; 50.6% were male, and the mean age was 6.8 years (range 5–10 years). Because written informed parental consent was required for participation, systematic sampling of children could not be applied as intended. All children with signed consent forms from parents/guardians were enrolled in the survey. One small private school (St. Theresa) was not sampled because of school officials refusal to participate. Demographic information was not available for 57 students from Tafuna Elementary. For 197 (17.4%) children enrolled, no blood sample was collected or the quantity of blood collected was insufficient for testing by ICT. Of the samples tested, 2/937 (0.2%, 95% upper confidence limit (CL) 0.8%) were antigen positive. Both positive children were from the same school, Lupelele Elementary. Demographic information and number of samples tested by ICT are summarized by school in Table 1.

**Table 1:** All public and private elementary schools in American Samoa included in TAS 1 and TAS 2. Age and sex distribution of children enrolled in TAS 1 and TAS 2 and number of samples tested for circulating filarial antigen by ICT are summarized by survey year.

	TAS 1 (February 2011)						TAS 2 (April 2015)					
School	Total enrollment in grades 1 and 2	Number enrolled	Number of males	Mean Age in years	Number tested by ICT	% tested by ICT	Total enrollment in grades 1 and 2	Number enrolled	Number of males	Mean Age in years	Number tested by ICT	% tested by ICT
A P Lutali	21	18	11	6.7	16	88.9	23	12	6	7.0	12	100.0
Afonotele	24	16	11	6.8	12	75.0	14	7	3	7.4	6	85.7
Alataua	76	40	19	7.0	38	95.0	64	32	15	6.7	29	90.6
Alofau	34	24	16	6.8	18	75.0	38	16	9	6.9	13	81.3
Aoa	30	27	16	6.7	26	96.3						
Aua	114	59	27	6.9	47	79.7	88	25	10	7.2	14	56.0
Coleman	210	71	36	6.7	63	88.7	143	41	16	7.2	37	90.2
Kanana Fou	35	10	3	6.7	10	100.0	82	45	19	6.8	40	88.9
Lauli'i	43	32	15	6.6	25	78.1	37	19	13	6.6	18	94.7
Le'atele							20	11	7	6.7	4	36.4
Leone Midkiff	269	130	77	6.6	113	86.9	195	76	45	7.0	76	100.0
Lupelele	203	106	60	6.8	92	86.8	167	87	37	7.0	85	97.7
Manulele	249	119	69	6.7	90	75.6	250	51	20	7.4	44	86.3
Manumalo	165	63	32	7.2	48	76.2	126	45	21	6.8	44	97.8
Masefau	19	16	7	6.6	13	81.3	21	13	5	7.3	9	69.2
Matafao	174	37	21	6.6	34	91.9	124	55	23	7.1	35	63.6
Matatula	38	14	8	6.8	14	100.0	39	26	16	6.8	26	100.0
Mt. Alava							18	12	6	6.8	10	83.3
Olomoana							19	7	6	7.4	6	85.7
Pacific Horizon							16	1	0	7.0	1	100.0
Pavaiai	294	158	83	6.5	107	67.7	278	96	49	6.9	89	92.7
Peteli							15	8	3	6.8	8	100.0
Samoa Baptist	38	4	1	7.0	3	75.0	35	13	7	7.5	12	92.3
SDA	32	17	9	6.9	11	64.7	18	12	4	7.0	10	83.3
Siliaga	59	31	19	7.0	28	90.3	41	16	7	6.8	14	87.5
South Pacific Academy	35	13	2	6.9	7	53.8	29	13	7	7.0	9	69.2
SPICC	78	30	14	6.6	25	83.3	66	27	17	7.1	27	100.0
St. Francis	41	29	12	6.7	28	96.6	29	10	4	7.0	10	100.0
St. Theresa	34						43	18	12	7.0	18	100.0
Tafuna	216	57	N/A	N/A	57	100.0	197	68	31	6.9	60	88.2
Ta'iala							11	2	0	8.0	2	100.0
Vatia	16	13	6	6.8	11	84.6						
**TOTALS**	**2,547**	**1,134**	**574**	**6.8**	**937**	**82.6**	**2,246**	**864**	**418**	**7.0**	**768**	**88.9**

### TAS 2

By 2015, two elementary schools that had existed in 2011 were closed and six new schools had opened. A total of 864 children from all 30 public and private elementary schools were enrolled in TAS 2; 48.4% were male, and the mean age was 7.0 years. As with TAS 1, written parental consent was required for participation and all children with signed consent forms were enrolled in the survey. For 96 (11.1%) children enrolled, no blood sample was collected or the quantity of blood collected was insufficient for ICT testing. Of the samples tested, 1/768 (0.1%, 95% CL 0.3%) was positive. The antigen-positive child was from Lupelele Elementary, the same school where the two antigen-positive children were identified in TAS 1 four years earlier. Demographic information and number of samples tested by ICT for TAS 2 are given in Table 1, stratified by school.

### Antibody responses

**Fig 1:**
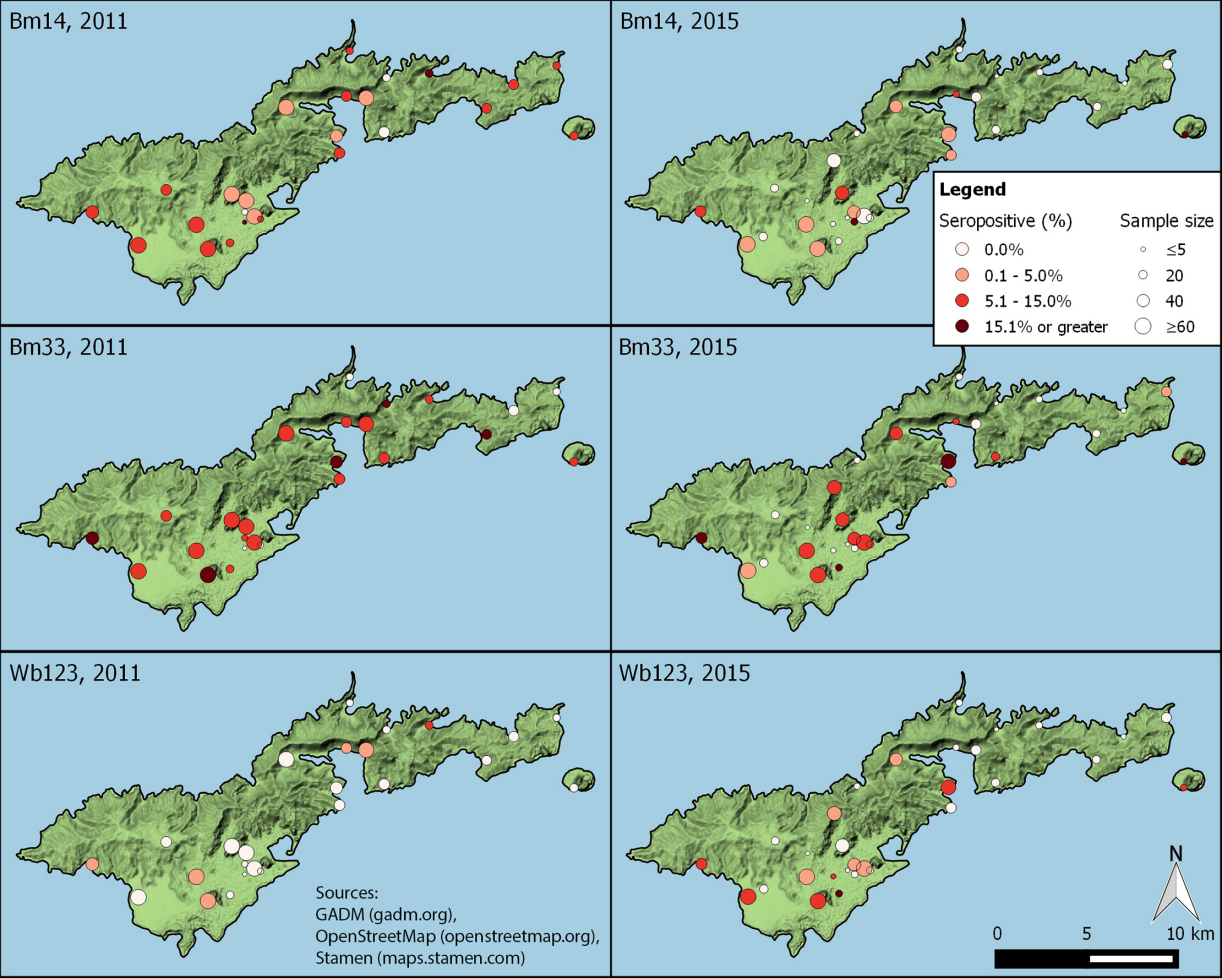
Distribution of antibody responses to Bm14, Bm33, and Wb123 by school for TAS 1 (2011) and TAS 2 (2015) in American Samoa. Responses to Wb123 in TAS 1 were assessed by luciferase immunoprecipitation system (LIPS) assay. All other responses were assessed by multiplex bead assay.

In 2011, a total of 1,112 DBS were prepared for antibody testing. Overall prevalence of responses to Wb123, Bm14, and Bm33 was 1.0%, 6.8% and 12.0%, respectively. There was at least one Wb123 antibody-positive child in 6/25 (24.0%) schools. Distribution of responses to Bm14 and Bm33 responses was more widespread than responses to Wb123 with at least one antibody-positive child identified in 88.0% and 80.0% of schools, respectively. In 2015, a total of 836 DBS were collected for antibody testing. Overall prevalence of Bm14 and Bm33 responses declined significantly to 3.0% (p<0.001) and 7.8% (p = 0.013), respectively. The prevalence of Wb123 responses was 3.6% in TAS 2, but results were not directly compared to those from TAS 1 because of the different testing platform used. The distribution of Wb123 responses was more widespread in TAS 2 than TAS 1 with at least one antibody-positive child identified in 12/30 (40.0%) schools. Distribution of Bm14 responses was more focal in 2015 than in 2011 with at least one antibody-positive child identified in 12/30 (40.0%) schools; only one of these 12 schools did not have any Bm14-positive children in TAS 1. Distribution of positive Bm33 responses was the most widespread (56.7% of schools) of the three markers assessed, but was still more focally distributed in 2015 compared to 2011. Antibody responses are summarized by school in Fig 1 and Table 2. Change in MFI-bg for Bm14 and Bm33 was compared for the 22 schools included in both surveys. There were significant declines in the median quantitative MBA responses for Bm14 and Bm33 in 21/22 (95.5%) schools. Median MFI-bg values are summarized in Table 3, stratified by school.

**Table 2:** Distribution of antibody responses to Wb123, Bm14, and Bm33 in all elementary schools located on the main island of Tutuila in American Samoa. Responses to Wb123 in TAS 1 were assessed by LIPS. All other responses were assessed by multiplex bead assay.

	TAS 1 (February 2011)							TAS 2 (April 2015)						
School	Number DBS* tested	Wb123 positive	% positive	Bm14 positive	% positive	Bm33 positive	% positive	Number DBS tested	Wb123 positive	% positive	Bm14 positive	% positive	Bm33 positive	% positive
A P Lutali	18	0	0.0	1	5.6	2	11.1	11	1	9.1	2	18.2	2	18.2
Afonotele	15	0	0.0	0	0.0	4	26.7	5	0	0.0	0	0.0	0	0.0
Alataua	40	2	5.0	5	12.5	8	20.0	31	3	9.7	4	12.9	6	19.4
Alofau	24	0	0.0	2	8.3	5	20.8	16	0	0.0	0	0.0	0	0.0
Aoa	27	0	0.0	4	14.8	0	0.0							
Aua	56	1	1.8	1	1.8	3	5.4	25	0	0.0	0	0.0	0	0.0
Coleman	70	0	0.0	2	2.9	8	11.4	40	1	2.5	2	5.0	3	7.5
Kanana Fou	10	0	0.0	0	0.0	1	10.0	41	1	2.4	2	4.9	3	7.3
Lauli'i	30	0	0.0	0	0.0	3	10.0	18	0	0.0	0	0.0	1	5.6
Le'atele								10	0	0.0	0	0.0	0	0.0
Leone Midkiff	129	0	0.0	10	7.8	12	9.3	75	4	5.3	1	1.3	2	2.7
Lupelele	105	5	4.8	15	14.3	27	25.7	85	5	5.9	1	1.2	11	12.9
Manulele	118	0	0.0	5	4.2	13	11.0	48	1	2.1	0	0.0	5	10.4
Manumalo	62	0	0.0	2	3.2	5	8.1	45	0	0.0	4	8.9	3	6.7
Masefau	16	1	6.3	3	18.8	2	12.5	12	0	0.0	0	0.0	0	0.0
Matafao	37	0	0.0	1	2.7	7	18.9	53	6	11.3	1	1.9	9	17.0
Matatula	14	0	0.0	1	7.1	0	0.0	25	0	0.0	0	0.0	1	4.0
Mt. Alava								12	0	0.0	0	0.0	0	0.0
Olomoana								7	0	0.0	0	0.0	0	0.0
Pacific Horizon								1	0	0.0	0	0.0	0	0.0
Pavaiai	155	1	0.6	11	7.1	13	8.4	94	4	4.3	4	4.3	9	9.6
Peteli								7	1	14.3	0	0.0	0	0.0
Samoa Baptist	4	0	0.0	1	25.0	0	0.0	12	0	0.0	2	16.7	0	0.0
SDA	15	0	0.0	1	6.7	1	6.7	12	2	16.7	0	0	2	16.7
Siliaga	29	0	0.0	2	6.9	3	10.3	16	0	0.0	0	0.0	0	0.0
South Pacific Academy	10	0	0.0	1	10.0	0	0.0	13	0	0.0	0	0.0	1	7.7
SPICC	29	0	0.0	2	6.9	4	13.8	27	0	0.0	0	0.0	1	3.7
St. Francis	28	1	3.6	4	14.3	4	14.3	10	0	0.0	1	10.0	1	10.0
St. Theresa								18	0	0.0	1	5.6	0	0.0
Tafuna	58	0	0.0	1	1.7	8	13.8	65	1	1.5	0	0.0	5	7.7
Ta'iala								2	0	0.0	0	0.0	0	0.0
Vatia	13	0	0.0	1	7.7	0	0.0							
**TOTALS**	**1112**	**11**	**1.0**	**76**	**6.8**	**133**	**12.0**	**836**	**30**	**3.6**	**25**	**3.0**	**65**	**7.8**

*DBS = dried blood spots

**Table 3:** Median fluorescence intensity minus background (MFI-bg) values by school for the 22 schools included in both TAS 1 (February 2011) and TAS 2 (April 2015) in American Samoa. Minimum and maximum MFI-bg values within each school are indicated in parentheses.

			Bm14			Bm33		
School	N, 2011	N, 2015	2011	2015	p	2011	2015	p
A P Lutali	18	11	45 (18, 379)	16 (4, 24,429)	0.001	347 (190, 1,611)	140 (12, 5,252)	<0.001
Afonotele	15	5	42 (20, 144)	16 (5, 23)	<0.001	329 (193, 3,452)	96 (58, 342)	0.039
Alataua	39	31	46 (23, 16,379)	14 (3, 25,424)	<0.001	451 (152, 26,090)	104 (12, 26,761)	<0.001
Alofau	24	16	46 (21, 333)	15 (1, 120)	<0.001	365 (109, 5,397)	90 (5, 381)	<0.001
Aua	56	25	39 (13, 274)	10 (2, 79)	<0.001	253 (108, 6,881)	58 (10, 251)	<0.001
Coleman	70	40	45 (16, 455)	12 (3, 7,014)	<0.001	244 (119, 16,449)	152 (71, 10,725)	<0.001
Kanana Fou	10	41	37 (23, 73)	10 (0, 2,162)	<0.001	169 (102, 1,273)	80 (-28, 30,402)	<0.001
Lauli'i	30	18	53 (20, 157)	20 (6, 148)	<0.001	401 (151, 2,992)	124 (54, 664)	<0.001
Leone Midkiff	128	75	50 (15, 872)	11 (-2, 844)	<0.001	454 (99, 5,373)	90 (-4, 24,291)	<0.001
Lupelele	103	85	43 (13, 24,135)	14 (5, 11,059)	<0.001	536 (107, 30,886)	158 (34, 4,563)	<0.001
Manulele	116	48	49 (16, 859)	14 (3, 150)	<0.001	384 (132, 4,033)	141 (15, 23,015)	<0.001
Manumalo	62	45	39 (13, 317)	18 (7, 11,800)	<0.001	288 (109, 4,137)	124 (37, 7,536)	<0.001
Masefau	16	12	54 (25, 761)	9 (2, 147)	<0.001	356 (118, 5,354)	139 (0, 219)	<0.001
Matafao	37	53	40 (20, 299)	13 (2, 15,237)	<0.001	272 (100, 8,988)	151 (-38, 24,914)	0.001
Matatula	14	25	39 (32, 172)	15 (7, 71)	<0.001	309 (127, 649)	114 (26, 1,652)	<0.001
Pavaiai	153	94	43 (9, 1,847)	13 (4, 25,471)	<0.001	304 (55, 15,131)	158 (-15, 14,071)	<0.001
Samoa Baptist	4	12	26 (17, 1,821)	14 (6, 4,680)	0.231	179 (167, 753)	68 (8, 244)	<0.001
SDA	15	12	51 (18, 3,644)	11 (4, 86)	0.002	282 (148, 8,449)	175 (74, 1,148)	0.377
Siliaga	29	16	42 (16, 300)	6 (2, 24)	<0.001	340 (139, 2,275)	68 (47, 355)	<0.001
South Pacific Academy	10	13	27 (13, 169)	11 (5, 35)	<0.001	216 (100, 495)	124 (80, 696)	0.001
SPICC	28	27	35 (14, 877)	19 (4, 360)	0.004	281 (97, 13,161)	83 (9, 1,477)	<0.001
St. Francis	28	10	44 (19, 5,180)	15 (5, 393)	<0.001	223 (105, 2,732)	131 (52, 904)	0.051

All three ICT-positive children identified in the surveys had positive antibody responses to Wb123, Bm14 and Bm33. However, concordance of individual antibody responses for antigen-negative children was relatively poor at both time points. Only 21/161 (13.0%) antibody-positive children in TAS 1 and 20/73 (27.4%) positive children in TAS 2 had positive responses to at least two markers. Similar discordance was also observed for samples tested where the antigen status was unknown. Test concordance for antigen negative children is summarized in Table 4. Test concordance for children whose antigen status was unknown is summarized in Table 5.

**Table 4:** Antibody test concordance among antigen negative children in TAS 1 (February 2011) and TAS 2 (April 2015) in American Samoa.

			Concordance with positive index		
	Index	total # positive/N (%)	Wb123	Bm14	Bm33
**TAS 1**	**Wb123**	7/935 (0.75)		6/7 (85.7)	7/7 (100.0)
	**Bm14**	63/935 (6.7)	6/63 (9.5)		20/63 (31.7)
	**Bm33**	118/935 (12.6)	7/118 (5.9)	20/118 (16.9)	
**TAS 2**	**Wb123**	26/743 (3.5)		11/26 (42.3)	16/26 (61.5)
	**Bm14**	22/743 (3.0)	11/22 (50.0)		15/22 (68.2)
	**Bm33**	56/743 (7.5)	16/56 (28.6)	15/56 (26.8)	

**Table 5:** Antibody test concordance among children with unknown antigen status in TAS 1 (February 2011) and TAS 2 (April 2015) in American Samoa.

			Concordance with positive index		
	Index	total # positive/N (%)	Wb123	Bm14	Bm33
**TAS 1**	**Wb123**	2/175 (1.1)		2/2 (100.0)	1/2 (50.0)
	**Bm14**	11/175 (6.3)	2/11 (18.2)		4/11 (36.4)
	**Bm33**	13/175 (7.4)	1/13 (7.7)	4/13 (30.8)	
**TAS 2**	**Wb123**	3/92 (3.3)		0/3 (0.0)	2/3 (66.7)
	**Bm14**	2/92 (2.2)	0/2 (0.0)		1/2 (50.0)
	**Bm33**	8/92 (8.7)	2/8 (25.0)	1/8 (12.5)	

## Discussion

The TAS is used to determine when transmission of LF is low enough that MDA can safely be stopped. The attraction of the TAS design is that it facilitates decision-making and has proven feasible to implement yet is standardized and incorporates a statistically rigorous design. After stopping MDA, WHO recommends repeating TAS twice at 2- to 3-year intervals and conducting additional surveillance activities to confirm that transmission has been interrupted. However, beyond the TAS, post-MDA surveillance guidance has not been standardized. A current need in the global effort to eliminate LF is reliable diagnostic tools that can be used to guide programmatic decisions, especially decisions made in the final stages of the program [22]. In principle, because of the greater sensitivity of detection of antibody responses compared to antigen testing, antibody testing could provide an earlier signal of recrudescence or decreased transmission over time. Antibody testing could be included in the TAS without any modification of the survey design. The inclusion of antifilarial antibody testing in both TAS 1 and TAS 2 conducted in American Samoa in 2011 and 2015, offered an opportunity to determine whether antibody testing would provide evidence that interruption of LF transmission had been achieved.

Results of TAS 1 conducted in 2011 met criteria for stopping MDA, and results from TAS 2 carried out in 2015 were below the threshold for which MDA was recommended. The outcomes from these surveys indicated that LF transmission had been reduced below the threshold at which transmission was thought to be sustainable. Although there was a decrease in the absolute number of antigen-positive children identified from TAS 1 to TAS 2, it was not possible to determine if antigen prevalence had changed between the two surveys since TAS is not statistically powered to detect changes over time. This illustrates a key challenge in post-MDA monitoring–as transmission declines and programs near elimination endpoints, it becomes increasingly difficult to rely on microfilariae (mf) and antigen markers.

Ideally, during the post-MDA surveillance period, trends could be measured to provide information on LF status in order to assist programs to take appropriate action as necessary. Since measures of antibody responses are more sensitive than mf and antigen detection [6], it is conceivable that changes in antibody prevalence could be used in the context of TAS to complement antigen testing. Although antigen prevalence in TAS 1 was <1%, responses to Wb123, Bm14, and Bm33 were 1.0%, 6.8%, and 12.0%, respectively. Similarly, antigen prevalence was low in TAS 2, but antibody prevalence was greater than antigen prevalence by all three markers. While there are limitations to using antigen markers during the surveillance period, the ability to monitor LF transmission status may be facilitated by using more sensitive antibody markers.

There were, however, differences among the three antibody markers. In TAS 1, the distribution of positive responses to Wb123 was relatively focal with antibody positive children in <25% of schools. In contrast, the distribution of positive Bm14 and Bm33 responses was more widespread with at least one antibody-positive child identified in >80% of schools. The more widespread distribution of Wb123 responses seen in TAS 2 may have been in part a function of using a different assay platform in 2015. Responses to Bm14 in TAS 2 were also fairly widespread, but less common than in TAS 1. However, the schools with Bm14-positive children were not necessarily the same ones in which Wb123-positive children were identified. Distribution of positive Bm33 responses was the most widespread of the three antibody markers assessed in TAS 2, detected in children in more than half of the schools, but was still more focally distributed than in TAS 1. It is possible that the three antibody markers used were measuring different LF exposure or infection patterns, but it is unclear how results of antibodies to a single marker should be interpreted.

There was good concordance of antibody responses among antigen-positive children. As expected, presence of CFA was associated with positive antibody responses to all three markers; Wb123, Bm14, and Bm33. However, among antigen-negative children, concordance of the antibody responses was poor, and a similar pattern was observed among children whose antigen status was unknown. These differences in antibody responses could be a reflection of the differences between antibody responses triggered by larval (Wb123) and adult worm (Bm14 and Bm33) exposures, but further research is needed to characterize LF antibody responses, especially during the post-MDA surveillance period.

In principle, as LF programs successfully implement MDA, reduced transmission of LF will result in lower prevalence of infection-specific antibody in young children and eventually an absence of detectable antibodies in the population. Overall, on the main island of American Samoa, there was a significant decline in antibody responses to Bm14 and Bm33 from 2011 to 2015 suggesting LF transmission was declining in the area. Furthermore, in every school included in both surveys, there was a significant decrease in the intensity of antibody responses from TAS 1 to TAS 2 providing additional support that LF exposure and infection had decreased during this period.

Although the overall decline in Bm14 and Bm33 prevalence suggested lower exposure and infection among young children, the results should be interpreted with caution. Positive antibody responses to these antigens were relatively widespread across the island in 2011. Even though responses to Bm14 and Bm33 were more focal in 2015, there were persistent responses in some schools. Furthermore, there was an apparent increase in Wb123 prevalence, but results of the two surveys could not be directly compared because of the different testing platforms used. Antibody responses in these schools could represent focal areas of persistent or recurrent LF transmission, residual seropositivity following interruption of transmission, or false-positive results. Bm14 and Bm33 are known to cross-react with closely related filarial parasites [23, 24], but these parasites are not known to be in circulation in American Samoa. It is also possible that the cutoff values for the MBA were inaccurate. The ability to define robust cutoffs for serological assays can be challenging and is often limited by the availability of well characterized panels of samples to determine appropriate cutoffs. If the responses represent residual seropositivity after interruption of transmission, then seroprevalence will continue to decline, and future surveys can be conducted to confirm the downward trend. While there may have been issues with defining antibody assay parameters, the possibility that persistent antibody signals represented true ongoing focal transmission cannot be excluded. Although the duration of antibody responses to Bm14 and Bm33 is unclear, presumably, positive signals among children in the target age range for TAS (6–7 years) indicate relatively recent exposure.

All three CFA-positive children identified in TAS 1 and TAS 2 were from the same school (Lupelele Elementary), and there were antibody-positive children in this school at both time points. These results may have been an indication of LF status in the communities in which the children lived. Yet, since the primary vector, *Aedes polynesiensis*, is a day-biting mosquito, it is difficult to determine if LF exposure took place in the community or elsewhere. In areas of diurnal LF periodicity, there is a need to better understand the relationship between results from school-based surveys and community transmission.

At present, it is unclear how to interpret persistent antibody responses in TAS 1 and TAS 2 in American Samoa. Although CFA results were below the threshold for which MDA was warranted, persistence of positive antibody signals is a potential cause for concern. In an island-wide vector study conducted in parallel to TAS 1, a large sample of mosquitoes was collected and tested for the presence of filarial DNA [25]. Results from the xenomonitoring survey indicated widespread, PCR-positive mosquitoes across the island. The presence of filarial DNA in mosquitoes raised suspicion that LF transmission was ongoing, but in the absence of established thresholds for programmatic action, no additional interventions were conducted. Additionally, in a serosurvey conducted just prior to TAS 1 there was evidence of possible clustering of antigen-positive adults in some communities [26]. Although the potential impact of antigen-positive clusters on transmission is unclear, it is possible that infection levels are high enough in these areas to sustain transmission. Furthermore, results from a recent study in American Samoa demonstrated that PCR-positive pools of LF vector mosquitoes were statistically significant predictors of seropositivity for Wb123 but not for Bm14 [27], suggesting Wb123 could be an indicator of ongoing transmission. Although Wb123 prevalence was low in both TAS, it may have been an indication of recent larval exposures and the potential for ongoing transmission. This also highlights the importance of understanding how to appropriately interpret responses to each of the antibody markers.

It is unclear why there was an apparent disconnect between declining antibody prevalence in TAS and evidence of ongoing transmission in other survey results. It is possible that the school-based cluster survey approach was not sensitive enough to reflect LF status in communities. Although all primary schools were included in the TAS, children from multiple villages attended each school. The transmission status of individual communities may have been more accurately reflected if a certain proportion of children from each village was tested. There is a need to better understand the significance of spatial distribution of CFA and antibody signals. Additionally, there may have been potential bias introduced in the way the TAS were conducted. The requirement for written informed consent resulted in lower than desired participation in surveys, and a true random sample of children could not be obtained. This highlights one of the challenges in areas where survey participation is dependent on intensive sensitization of communities.

Currently, there is no clear guidance on how to investigate CFA- or antibody-positive children identified during TAS, but utilizing survey results may allow programs to identify areas where additional interventions may be needed. In American Samoa, there was an overall decline in antibody prevalence, but there were indications that raised concerns of ongoing transmission. All antigen-positive children were from the same school, but no specific follow up activities took place. Furthermore, there were schools in which there were persistent antibody signals from TAS 1 to TAS 2, which may have been an indication of focal transmission. There are challenges in utilizing cluster-based survey methodologies to assess focal diseases especially when prevalence is assumed to be low. There have been activities conducted in Sri Lanka to determine relationships among various indicators, including CFA, antifilarial antibody, and the presence of filarial DNA in *Culex* mosquitoes [28, 29]. However, there is a pressing need to conduct similar activities in *Aedes* spp. and *Anopheles* spp. settings.

As GPELF continues to make progress, it is critical to identify strategies for effective monitoring and evaluation to determine if transmission has been interrupted. Making incorrect programmatic decisions can have major political and financial implications. Despite passing TAS 1 and TAS 2, clustering and persistence of positive antibody responses in schools may be an indication of ongoing transmission in American Samoa. Although there is a clear need to better understand the limitations of current antibody tests, our results suggest that serologic tools can have a role in guiding programmatic decision making.

### Disclaimer

The findings and conclusions in this report are those of the authors and do not necessarily represent the official position of the Centers for Disease Control and Prevention.

## Supporting information

S1 Checklist STROBE checklist. (DOC)
