# Supplementary material for: Comparison of antigen and antibody responses in repeat lymphatic filariasis transmission assessment surveys in American Samoa
Source: PLoS Negl Trop Dis. 2018 Mar 9;12(3):e0006347. doi: 10.1371/journal.pntd.0006347 (PMC5862496; doi:10.1371/journal.pntd.0006347)
Supplement: S1 Checklist — (DOC) [file pntd.0006347.s001.doc]

STROBE Statement—Checklist of items that should be included in reports of ***cross-sectional studies***

|  | Item No | Recommendation |
| --- | --- | --- |
| **Title and abstract** | 1 | (*a*) Indicate the study’s design with a commonly used term in the title or the abstract  **The title includes “lymphatic filariasis transmission assessment surveys” which is a WHO-recommended survey for LF programs. Survey methodology can be found in official WHO documents.** |
|  |  | (*b*) Provide in the abstract an informative and balanced summary of what was done and what was found  **page 2, lines 21-33** |
| Introduction | | |
| Background/rationale | 2 | Explain the scientific background and rationale for the investigation being reported  **page 6, lines 82-92** |
| Objectives | 3 | State specific objectives, including any prespecified hypotheses  **page 7, lines 104-108** |
| Methods | | |
| Study design | 4 | Present key elements of study design early in the paper  **page 7, line 125; page 8, lines 126-133** |
| Setting | 5 | Describe the setting, locations, and relevant dates, including periods of recruitment, exposure, follow-up, and data collection  **page 7, lines 121-125** |
| Participants | 6 | (*a*) Give the eligibility criteria, and the sources and methods of selection of participants  **page 8, lines 127-130** |
| Variables | 7 | Clearly define all outcomes, exposures, predictors, potential confounders, and effect modifiers. Give diagnostic criteria, if applicable  **page 8, lines 131-146; page 9, lines 147-160** |
| Data sources/ measurement | 8* | For each variable of interest, give sources of data and details of methods of assessment (measurement). Describe comparability of assessment methods if there is more than one group  **page 8, lines 131-147; page 9, lines 148-163** |
| Bias | 9 | Describe any efforts to address potential sources of bias  **page 8, lines 129-130** |
| Study size | 10 | Explain how the study size was arrived at  **page 8, lines 126-127; 130-131** |
| Quantitative variables | 11 | Explain how quantitative variables were handled in the analyses. If applicable, describe which groupings were chosen and why  **page 10, lines 169-175** |
| Statistical methods | 12 | (*a*) Describe all statistical methods, including those used to control for confounding  **page 10, lines 169-175** |
|  |  | (*b*) Describe any methods used to examine subgroups and interactions  **page 10, lines 169-175** |
|  |  | (*c*) Explain how missing data were addressed  **page 10, lines 169-175** |
|  |  | (*d*) If applicable, describe analytical methods taking account of sampling strategy  **page 10, lines 169-175** |
|  |  | (*e*) Describe any sensitivity analyses  **N/A** |
| Results | | |
| Participants | 13* | (a) Report numbers of individuals at each stage of study—eg numbers potentially eligible, examined for eligibility, confirmed eligible, included in the study, completing follow-up, and analysed  **page 10, lines 178; page 13, lines 195; Table 1** |
|  |  | (b) Give reasons for non-participation at each stage  **page 10, lines 180-185; page 13, lines 196-199** |
|  |  | (c) Consider use of a flow diagram  **N/A** |
| Descriptive data | 14* | (a) Give characteristics of study participants (eg demographic, clinical, social) and information on exposures and potential confounders  **page 10, lines 178-179; page 13, lines 195-196; Table 1** |
|  |  | (b) Indicate number of participants with missing data for each variable of interest  **page 10, lines 183-185; page 13, lines 198-199** |
| Outcome data | 15* | Report numbers of outcome events or summary measures  **page 10, line 186; page 13, lines 200; 205-214; page 14, lines 212-220; page 17, lines 234-241; Tables 2, 3, 4, 5** |
| Main results | 16 | (*a*) Give unadjusted estimates and, if applicable, confounder-adjusted estimates and their precision (eg, 95% confidence interval). Make clear which confounders were adjusted for and why they were included  **page 10, line 183; page 12, line 197; page 13, lines 202-211; page 14, lines 215-222; page 17, lines 237-242; Tables 2, 3, 4, 5** |
|  |  | (*b*) Report category boundaries when continuous variables were categorized  **N/A** |
|  |  | (*c*) If relevant, consider translating estimates of relative risk into absolute risk for a meaningful time period  **N/A** |
| Other analyses | 17 | Report other analyses done—eg analyses of subgroups and interactions, and sensitivity analyses  **N/A** |
| Discussion | | |
| Key results | 18 | Summarise key results with reference to study objectives  **pages 18-24, lines 257-394** |
| Limitations | 19 | Discuss limitations of the study, taking into account sources of potential bias or imprecision. Discuss both direction and magnitude of any potential bias  **page 21, lines 326-332; page 22, lines 342-345; page 23, lines 364-385; page 24, lines 386-387** |
| Interpretation | 20 | Give a cautious overall interpretation of results considering objectives, limitations, multiplicity of analyses, results from similar studies, and other relevant evidence  **pages 24, lines 388-394** |
| Generalisability | 21 | Discuss the generalisability (external validity) of the study results  **page 19, lines 281-290; page 20, lines 312-319; pages 23-24, lines 389-394** |
| Other information | | |
| Funding | 22 | Give the source of funding and the role of the funders for the present study and, if applicable, for the original study on which the present article is based  **Pages 25, lines 408-414** |

*Give information separately for exposed and unexposed groups.

**Note:** An Explanation and Elaboration article discusses each checklist item and gives methodological background and published examples of transparent reporting. The STROBE checklist is best used in conjunction with this article (freely available on the Web sites of PLoS Medicine at http://www.plosmedicine.org/, Annals of Internal Medicine at http://www.annals.org/, and Epidemiology at http://www.epidem.com/). Information on the STROBE Initiative is available at www.strobe-statement.org.
